# Practical Departments

**Published:** 1905-03-04

**Authors:** 


### PRACTICAL DEPARTMENTS.

### A COTTAGE HOSPITAL OF FOUR OR SIX BEDS.

A correspondent writes: We have in contemplation a cottage hospital of four or six beds for this village and should feel very grateful if you would kindly help us to form an estimate of the up-keep of such an establishment.

[Our correspondent should procure a copy of "Cottage Hospitals," Third Edition, published by The Scientific Press, 28 Southampton Street, Strand, London, where all the information required is given in the simplest and most understandable form. There is also a small pamphlet on the subject, entitled, "The Cottage Hospital," published by the same firm.—Ed. The Hospital.]

